# Supplementary figures and images for: Increased temperatures may safeguard the nutritional quality of crops under future elevated CO 2 concentrations
Source: Plant J. 2019 Jan 18;97(5):872–86. doi: 10.1111/tpj.14166 (PMC6850270; doi:10.1111/tpj.14166)

Figure S1

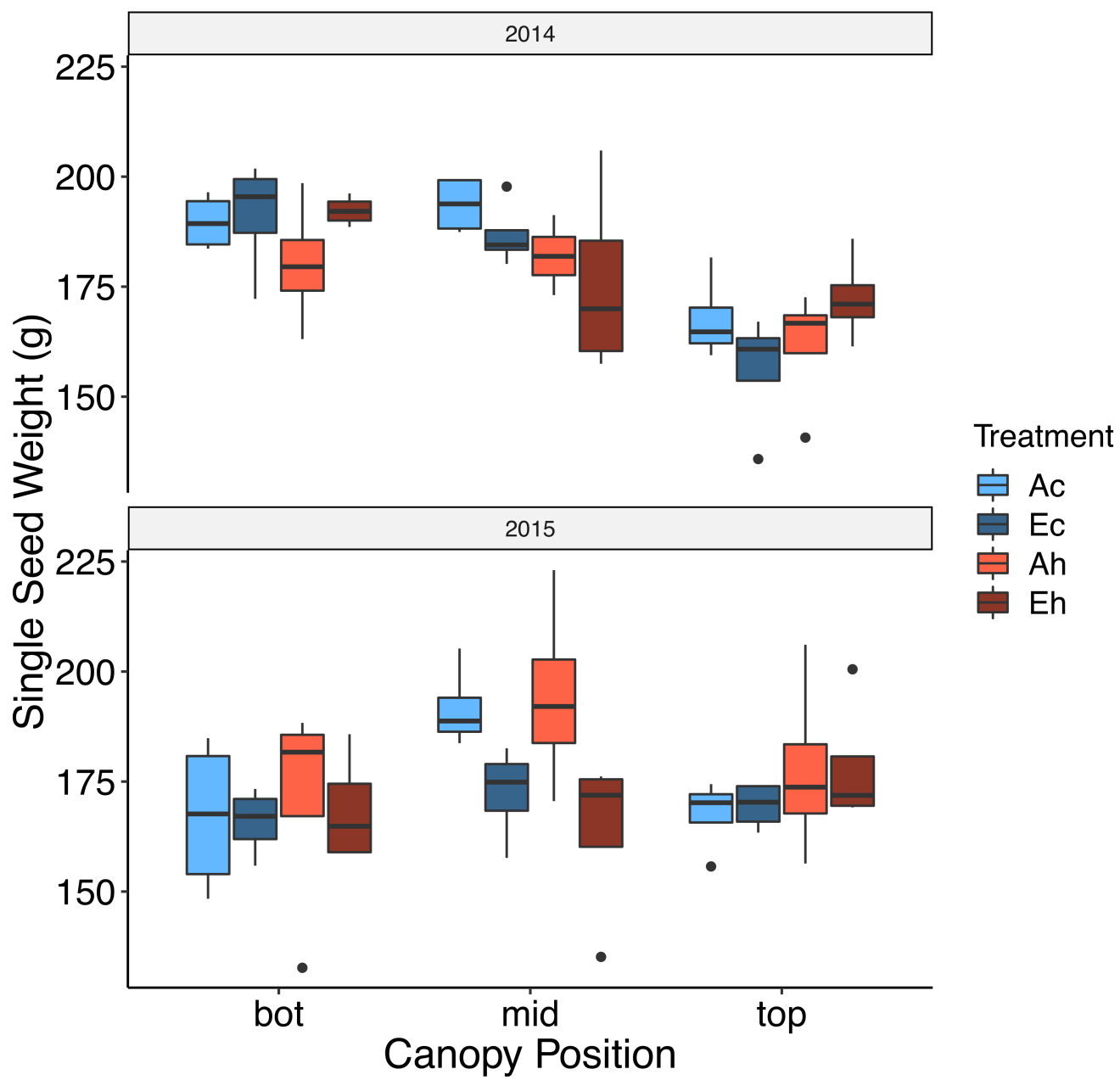

Supplement: Supplementary file 1 — Figure S1. Variation in single seed weight as a function of canopy position (bottom, middle or top third of the main stem) where the seeds were produced. A.Control, ambient CO2, control temperature; A.Hot, ambient CO2, heated + 3.5°C; E.Control, elevated CO2, control temperature; E.Hot, elevated CO2, heated + 3.5°C. [file TPJ-97-872-s001.pdf]

Figure S2

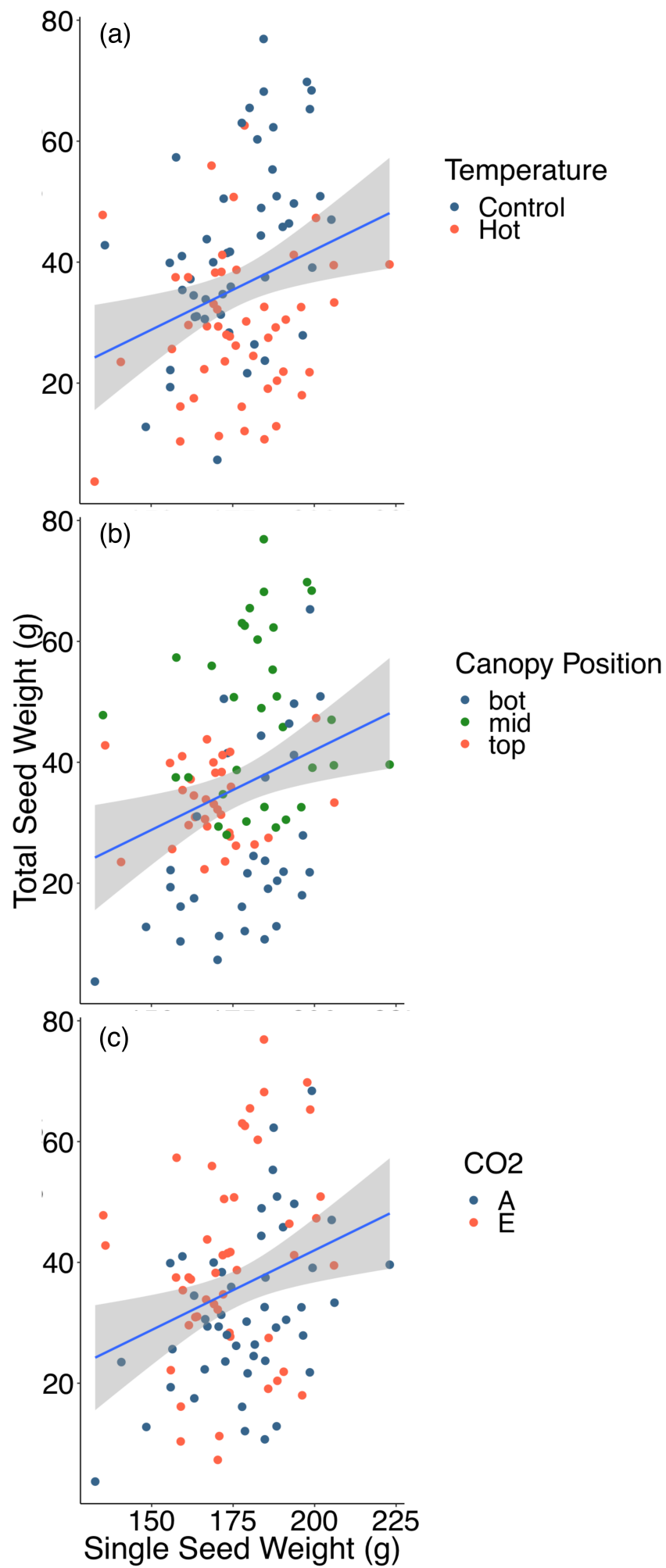

Supplement: Supplementary file 2 — Figure S2. Correlation plots between single seed weight and seed yield for the corresponding canopy position. Results from 2014 and 2015 were combined, and data points are color coded according to (a) ambient versus elevated temperature; (b) canopy position; and (c) ambient versus elevated CO2. The correlations were not statistically significant (P = 0.28) and simply document that changes in yield were primarily driven by variation in number of seeds produced. [file TPJ-97-872-s002.pdf]
