# Supplementary material for: Increased temperatures may safeguard the nutritional quality of crops under future elevated CO 2 concentrations
Source: Plant J. 2019 Jan 18;97(5):872–86. doi: 10.1111/tpj.14166 (PMC6850270; doi:10.1111/tpj.14166)
Supplement: Supplementary file 3 [file TPJ-97-872-s003.docx]

# Full legends for Supplemental Figures

**Figure S1.** Variation in single seed weight as a function of canopy position (bottom, middle or top third of the main stem) where the seeds were produced. A.Control, ambient CO_2_, control temperature; A.Hot, ambient CO_2_, heated + 3.5 °C; E.Control, elevated CO_2_, control temperature; E.Hot, elevated CO_2_, heated + 3.5 °C.

**Figure S2.** Correlation plots between single seed weight and seed yield for the corresponding canopy position. Results from 2014 and 2015 were combined and data points are color coded according to (a) ambient vs elevated temperature; (b) canopy position; and (c) ambient vs elevated CO_2_. The correlations were not statistically significant (p = 0.28) and simply document that changes in yield were primarily driven by variation in number of seeds produced.
